# Prevalence of Overweight, Obesity, and Abdominal Obesity and Their Associated Factors in Duhok City, Kurdistan Region, Iraq: A Population‐Based Cross‐Sectional Study

**DOI:** 10.1155/jobe/5599283

**Published:** 2026-09-30

**Authors:** Nawfal R. Hussein, Dildar H. Musa, Halder J. Abozait, Hiwa F. Abdulraheem, Delovan S. Mahfodh, Waleed M. Mohammed, Halat S. Majed, Ibrahim A. Naqid

**Affiliations:** ^1^ Department of Biomedical Sciences College of Medicine University of Zakho Zakho Independent Administration Zakho Kurdistan Region Iraq uoz-krg.org; ^2^ Department of Surgery College of Medicine University of Duhok Duhok Kurdistan Region Iraq uod.ac; ^3^ Department of Internal Medicine College of Medicine University of Duhok Duhok Kurdistan Region Iraq uod.ac; ^4^ Duhok Center for Diabetes and Endocrine Diseases Duhok Kurdistan Region Iraq

## Abstract

**Background:**

Obesity has become a global health epidemic, contributing significantly to numerous chronic diseases, including metabolic disorders, cardiovascular diseases, and certain cancers. This study aimed to assess the prevalence of overweight, obesity, and abdominal obesity and the associated factors in Duhok City, Kurdistan Region of Iraq.

**Methods:**

A population‐based cross‐sectional study was conducted in Duhok City, Iraq, including 490 participants aged 18 years and older. A multistage sampling technique was used, and data were collected via structured questionnaires covering sociodemographic and clinical characteristics. Anthropometric measurements were used to classify participants according to their body mass index (BMI) and waist circumference.

**Results:**

The overall prevalence of overweight, obesity, and abdominal obesity was 38.4%, 24.3%, and 72.9%, respectively. Factors significantly associated with overweight in multivariate logistic regression included age group 30–44 (adjusted odds ratio [aOR]: 2.356, *p* = 0.018), married individuals (aOR: 2.040, *p* = 0.038), and smokers (aOR: 0.562, *p* = 0.045). Obesity was associated with age group 30–44 (aOR: 2.769, *p* = 0.030) and age group 45–59 (aOR: 5.384, *p* = 0.002). Abdominal obesity was significantly associated with gender, where males had lower odds compared to females (aOR: 0.248, *p* = <0.001), age group 30–44 (aOR: 2.630, *p* = 0.011), age group 45–59 (aOR: 4.793, *p* = 0.002), married individuals (aOR: 3.313, *p* = 0.001), and sleep disturbance (aOR: 1.790, *p* = 0.025).

**Conclusions:**

The findings indicate a high prevalence of overweight, obesity, and abdominal obesity in Duhok City, with various associated factors. Public health initiatives targeting these associated factors are essential to address the growing obesity epidemic in the region. Further studies with broader geographic coverage and larger sample sizes are needed to reinforce these results and inform policy interventions.

## 1. Introduction

Overnutrition, resulting in overweight and obesity, has become a global pandemic, with an ongoing trend of rising prevalence over the past 2 decades, culminating in the term “globesity” to describe the global rise in obesity prevalence [1, 2]. Findings from the World Obesity Atlas 2025 indicate that we are missing the 2025 World Health Organization (WHO) target to stop the rise in obesity and project that, by 2030, over 2.9 billion adults will have a high body mass index (BMI), including 1.1 billion living with obesity [3]. Obesity is associated with several conditions, including type 2 diabetes, dyslipidemia, cardiovascular diseases, strokes, dementia, gallstones, fatty liver, osteoarthritis, hypoventilation syndrome, sleep apnea, and a wide range of cancers [4]. Obesity‐related mortality is well established and mainly due to vascular disease. Median survival is reduced by 2–4 years at a BMI of 30–35 kg/m^2^, and 8–10 years at a BMI of 40–45 kg/m^2^ (morbid obesity), comparable to the effects of smoking [5]. The number of premature mortalities from noncommunicable diseases due to obesity supersedes road traffic mortality [3].

Obesity results from an imbalance between energy intake and expenditure, influenced by multiple factors. Genetic factors could be monogenic, polygenic, or syndromic. Environmental factors include sedentary lifestyles and overnutrition, particularly unhealthy carbohydrates, which are increasingly prevalent due to urbanization. Obesity can also be caused by medical conditions such as Cushing’s syndrome and certain medications [6]. Childhood obesity is also a serious public health challenge, with prevalence rising in low‐ and middle‐income countries (LMICs) while remaining persistently high in many high‐income regions [7]. Most countries have either one or no policies in action to overcome the burden of obesity [3]. Recognizing these trends is essential for creating effective healthcare strategies and policies. Most obesity research in Iraq has concentrated on specific population groups or is outdated. In a sample of women from outpatient clinics in Baghdad in 2003, 39% were overweight and 25% were obese [8]. Among university students in 2011, 35.5% were overweight, while 29% were obese [9]. A different study focusing on preschool children in 2020 found that 14.8% were overweight and 5.9% were obese [10]. A national survey based on data from 2015 found that 31.8% were overweight and 33.9% had obesity [11].

Despite the existence of several studies and national data on the prevalence of overweight and obesity in Iraq, a substantial gap exists in prevalence estimates at the provincial level. Hence, province‐level evidence remains scarce, limiting our ability to clearly understand the disparity in the epidemiological patterns and trends of overweight and obesity across different Iraqi provinces. Additionally, national data may mask geographical variations and may, therefore, provide an incomplete picture of the local burden of overweight, obesity, and abdominal obesity. Furthermore, no recent population‐based studies investigating the prevalence of overweight, obesity, and their associated factors are available at the level of Duhok province. This data gap at the provincial level represents a major barrier to advocating for targeted public health initiatives tailored to the provincial needs. Therefore, this population‐based study aims to determine the prevalence of overweight, obesity, and abdominal obesity and their associated factors among adults in Duhok City, Kurdistan Region of Iraq.

## 2. Materials and Methods

### 2.1. Study Design and Population

A population‐based cross‐sectional study was conducted in Duhok City, Kurdistan Region of Iraq, between February 20 and March 1, 2025. Adults aged 18 years and older residing in Duhok City were eligible to participate. Pregnant women, subjects younger than 18 years old, or those who disagreed to participate were excluded. Moreover, questionnaires with missing information or incomplete data were excluded from the study. The study was conducted in accordance with the Strengthening the Reporting of Observational Studies in Epidemiology (STROBE) guidelines.

### 2.2. Sampling Strategy

A multistage sampling method was employed. Initially, the Duhok city center was divided into 12 districts based on geographical boundaries, with approximately equal population sizes across the districts. Six of the 12 districts were subsequently selected to ensure adequate geographical representation, while maintaining the feasibility of the study within the available capacity and resources. Additionally, in order to minimize selection bias, the 6 districts were selected using a random sampling technique, whereby each district had an equal probability of selection. Within each selected district, the starting household was identified using a computer‐generated random number. Thereafter, systematic sampling was employed, with every third household following the previously selected household being reached for participation. In each selected household, the first eligible participant who came into contact with the data collection team was invited to participate in the study.

### 2.3. Sample Size

The sample size was calculated using the formula for estimating population proportions, assuming *p* = 0.5, a 95% confidence interval (90% CI), and a 5% margin of error, resulting in a minimum sample size of 384. A total of 490 individuals were ultimately included. Figure 1 illustrates the participant recruitment process.

**FIGURE 1:**
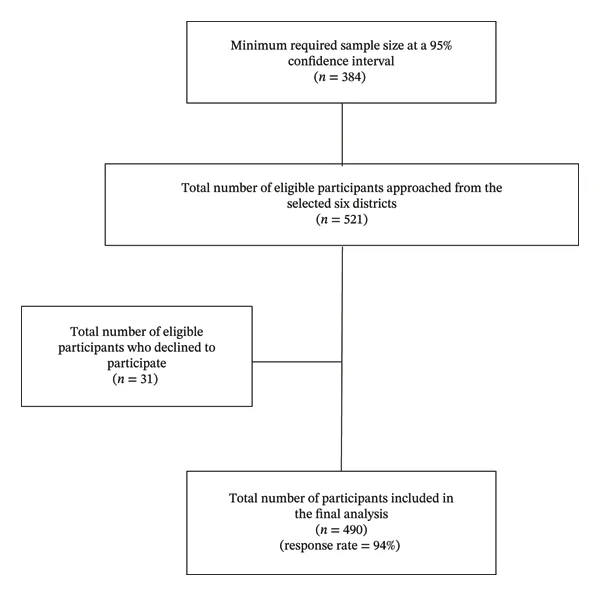
Flowchart for participant recruitment.

### 2.4. Data Collection

Data were collected using a structured questionnaire, consisting of participants’ demographic characteristics, smoking status, physical activity, and medical history. Data collection was conducted by 10 teams, each consisting of 3–5 trained medical students equipped with standardized anthropometric measurement devices. However, before data collection, experts in public health, community medicine, and research methodology provided standardized training to the data collection teams to ensure the standard and accurate administration of the questionnaire, consistent application of the study variable definitions, and accurate assessment of anthropometric measurements. In particular, the teams received practical guidance on the proper use, calibration, and handling of anthropometric measurement devices. These measures were taken to minimize any error associated with data collection and to enhance the reliability and quality of the collected data.

### 2.5. Clinical Definitions

Participants who had completed middle school, high school, or university education were classified as educated, whereas those who were illiterate or had only completed primary school were classified as uneducated. Physical activity was defined as engagement in any moderate or vigorous bodily movement such as walking, sports, or occupational activity for at least 150 min per week. Sleep disturbance was defined as self‐reported difficulty in initiating sleep, maintaining sleep, early morning awakening, or nonrestorative sleep occurring at least three nights per week during the previous month. Individuals who reported smoking during the interview were classified as smokers. Hypertension was defined as a self‐reported physician diagnosis or current use of antihypertensive medications. Diabetes mellitus was defined as a previous physician diagnosis or use of antidiabetic medication. Hyperlipidemia was defined as a self‐reported physician diagnosis or current use of lipid‐lowering medication.

### 2.6. Anthropometric Measurements

Weight and height were measured by trained staff using standardized equipment with a reliable weighing scale and a wall‐mounted tape measure in a standing position, respectively. BMI was calculated as weight (kg) divided by height squared (m^2^). Participants were classified according to WHO criteria: underweight (< 18.5 kg/m^2^), normal weight (18.5–24.9 kg/m^2^), overweight (25–29.9 kg/m^2^), and obesity (≥ 30 kg/m^2^) [1]. Waist circumference was measured with a plastic tape measure at the midpoint between the lowest rib and the iliac crest. Abdominal obesity was defined as waist circumference > 94 cm for men and > 80 cm for women [12].

### 2.7. Statistical Analysis

Data were entered and cleaned using Microsoft Excel and subsequently analyzed using IBM SPSS Statistics for Windows (Version 25.0; IBM Corp., Armonk, NY, USA). Descriptive statistics were used to summarize participants’ characteristics. Continuous variables were presented as mean ± standard deviation (SD), while categorical variables were expressed as frequencies and percentages. The prevalence of overweight, obesity, and abdominal obesity was calculated according to standard WHO criteria. Associations between categorical independent variables and weight status categories were initially assessed using the chi‐square test. Binary logistic regression analysis was then performed to estimate crude and adjusted odds ratios (aORs) with 95% CI for factors associated with overweight, obesity, and abdominal obesity. The variance inflation factor (VIF) was used to assess multicollinearity among the independent variables. All VIF values were < 5, indicating the absence of significant multicollinearity. Additionally, the goodness of fit of the multivariable logistic regression models for overweight, obesity, and abdominal obesity was assessed using the Hosmer–Lemeshow test. For all three models, the test was not statistically significant, indicating no evidence of poor or inadequate model fit. A *p* value of < 0.05 was considered statistically significant for all analyses.

### 2.8. Ethics

The Research and Ethics Committee at the College of Medicine, Zakho University in the Kurdistan Region of Iraq, granted approval for the study protocol and informed consent (JAN2025/UOZE40). Participation was entirely voluntary, and consent was acquired from all participants involved in the study. Moreover, data were mainly used for research purposes, and data anonymity and confidentiality were strictly maintained. The Declaration of Helsinki ethical standards for medical research involving human subjects were followed.

## 3. Results

### 3.1. Demographic Data

A total of 490 participants, including 281 (57.3%) males and 209 (42.7%) females, were included in the analysis. Their age ranged from 18 to 98 years, with a mean age of 38.6 ± 15.46. About three‐quarters (73.7%) of the participants were educated, 56.9% were married, and 54.5% were employed. Sociodemographic and clinical characteristics of the study population are shown in Table 1.

**TABLE 1:** Sociodemographic and clinical characteristics of study participants.

Variables	Female *n*. (%)	Male *n*. (%)	Total *n*. (%)
Age (years)			
18–29	84 (40.2%)	86 (30.6%)	170 (34.7%)
30–44	65 (31.1%)	93 (33.1%)	158 (32.2%)
45–59	48 (23%)	66 (23.5%)	114 (23.3%)
> 60	12 (5.7%)	36 (12.8%)	48 (9.8%)
Education status			
Uneducated	54 (25.8%)	75 (26.7%)	129 (26.3%)
Educated	155 (74.2%)	206 (73.3%)	361 (73.7%)
Marital status			
Single	109 (52.2%)	102 (36.3%)	211 (43.1%)
Married	100 (47.8%)	179 (63.7%)	279 (56.9%)
Employment status			
Unemployed	135 (64.6%)	88 (31.3%)	223 (45.5%)
Employed	74 (35.4%)	193 (68.7%)	267 (54.5%)
Smoking status			
Nonsmoker	201 (96.2%)	135 (48%)	336 (68.6%)
Smoker	8 (3.8%)	146 (52%)	154 (31.4%)
Physical activity			
No	160 (76.6%)	181 (64.4%)	341 (69.6%)
Yes	49 (23.4%)	100 (35.6%)	149 (30.4%)
Sleep disturbance			
No	116 (55.5%)	183 (65.1%)	299 (61%)
Yes	93 (44.5%)	98 (34.9%)	191 (39%)
Waist circumference			
Normal	35 (16.7%)	98 (34.9%)	133 (27.1%)
High	174 (83.3%)	183 (65.1%)	357 (72.9%)
BMI			
Underweight	7 (3.3%)	8 (2.8%)	15 (3.1%)
Normal	70 (33.5%)	98 (34.9%)	168 (34.3%)
Overweight	65 (31.1%)	123 (43.8%)	188 (38.4%)
Obese	67 (32.1%)	52 (18.5%)	119 (24.3%)

### 3.2. Prevalence of Overweight, Obesity, and Abdominal Obesity and Their Associated Factors

Following the WHO classification of BMI, 3.1% of the study participants were underweight, and 34.3% had a normal BMI. The overall prevalences of overweight and obesity were 38.4% (95% CI: 34.0%–42.8%) and 24.3% (95% CI: 20.6%–28.3%), respectively, while the prevalence of abdominal obesity was 72.9% (95% CI: 68.7%–76.8%). Different factors were analyzed for potential association with overweight, obesity, and abdominal obesity. The following factors were found to be significantly associated with overweight in multivariate logistic regression, including age group 30–44 (aOR: 2.356, *p* = 0.018), married individuals (aOR: 2.040, *p* = 0.038), and smokers (aOR: 0.562, *p* = 0.045). Obesity was associated with age group 30–44 (aOR: 2.769, *p* = 0.030) and age group 45–49 (aOR: 5.384, *p* = 0.002). Abdominal obesity was significantly associated with male gender (aOR: 0.248, *p* = <0.001), age group 30–44 (aOR: 2.630, *p* = 0.011), age group 45–49 (aOR: 4.793, *p* = 0.002), married individuals (aOR: 3.313, *p* = 0.001), and sleep disturbance (aOR: 1.790, *p* = 0.025). The complete results of univariate and multivariate logistic regression of factors associated with overweight, obesity, and abdominal obesity are presented in Tables 2, 3, and 4.

**TABLE 2:** Univariate and multivariate logistic regression of factors associated with overweight.

Factors	Category	Crude OR (95% CI)	*p* value	Adjusted OR (95% CI)	*p* value
Gender	Female	Reference		Reference	
	Male	1.352 (0.88–2.077)	0.169	1.248 (0.686–2.271)	0.467

Age (years)	18–29	Reference		Reference	
	30–44	3.732 (2.231–6.243)	< 0.001	2.356 (1.159–4.791)	0.018
	45–59	3.490 (1.829–6.660)	< 0.001	2.183 (0.883–5.397)	0.091
	≥ 60	3.878 (1.832–8.206)	< 0.001	2.871 (0.974–8.463)	0.056

Education status	Uneducated	Reference		Reference	
	Educated	0.836 (0.507–1.380)	0.484	1.656 (0.866–3.167)	0.127

Marital status	Single	Reference		Reference	
	Married	3.487 (2.254–5.397)	< 0.001	2.040 (1.039–4.007)	0.038

Employment status	Unemployed	Reference		Reference	
	Employed	1.868 (1.226–2.848)	0.004	1.105 (0.624–1.957)	0.732

Smoking status	Nonsmokers	Reference		Reference	
	Smokers	0.742 (0.481–1.147)	0.179	0.562 (0.319–0.988)	0.045

Physical activity	No	Reference		Reference	
	Yes	0.847 (0.643–1.323)	0.466	0.753 (0.457–1.241)	0.266

Sleep disturbance	No	Reference		Reference	
	Yes	0.688 (0.448–1.055)	0.087	0.951 (0.583–1.550)	0.839

Diabetes	No	Reference		Reference	
	Yes	1.994 (0.968–4.106)	0.061	1.242 (0.530–2.910)	0.618

Hypertension	No	Reference		Reference	
	Yes	1.462 (0.687–3.109)	0.324	0.856 (0.343–2.137)	0.739

Hyperlipidemia	No	Reference		Reference	
	Yes	1.990 (0.939–4.216)	0.073	1.250 (0.525–2.975)	0.614

Ischemic heart disease	No	Reference		Reference	
	Yes	2.026 (0.689–5.956)	0.199	1.167 (0.333–4.093)	0.809

**TABLE 3:** Univariate and multivariate logistic regression of factors associated with obesity.

Factors	Category	Crude OR (95% CI)	*p* value	Adjusted OR (95% CI)	*p* value
Gender	Female	Reference		Reference	
	Male	0.554 (0.345–0.891)	0.015	0.497 (0.237–1.044)	0.065

Age (years)	18–29	Reference		Reference	
	30–44	5.789 (2.906–11.533)	< 0.001	2.769 (1.101–6.962)	0.030
	45–59	16.625 (7.967–34.69)	< 0.001	5.384 (1.901–15.247)	0.002
	≥ 60	3.654 (1.307–10.211)	0.013	1.837 (0.482–7.009)	0.373

Education status	Uneducated	Reference		Reference	
	Educated	0.389 (0.231–0.656)	< 0.001	0.878 (0.414–1.864)	0.735

Marital status	Single	Reference		Reference	
	Married	6.133 (3.601–10.447)	< 0.001	2.251 (0.976–5.192)	0.057

Employment status	Unemployed	Reference		Reference	
	Employed	1.968 (1.220–3.174)	0.006	1.725 (0.828–3.593)	0.145

Smoking status	Nonsmokers	Reference		Reference	
	Smokers	0.411 (0.240–0.705)	0.001	0.524 (0.247–1.112)	0.092

Physical activity	No	Reference		Reference	
	Yes	0.686 (0.408–1.153)	0.155	0.829 (0.439–1.567)	0.564

Sleep disturbance	No	Reference		Reference	
	Yes	0.763 (0.472–1.235)	0.272	1.135 (0.620–2.080)	0.681

Diabetes	No	Reference		Reference	
	Yes	2.948 (1.396–6.227)	0.005	1.110 (0.433–2.846)	0.829

Hypertension	No	Reference		Reference	
	Yes	3.815 (1.844–7.895)	< 0.001	1.376 (0.546–3.470)	0.498

Hyperlipidemia	No	Reference		Reference	
	Yes	4.189 (1.985–8.839)	< 0.001	2.213 (0.874–5.599)	0.094

Ischemic heart disease	No	Reference		Reference	
	Yes	2.350 (0.749–7.369)	0.143	0.877 (0.195–3.954)	0.865

**TABLE 4:** Univariate and multivariate logistic regression of factors associated with abdominal obesity.

Factors	Category	Crude OR (95% CI)	*p* value	Adjusted OR (95% CI)	*p* value
Gender	Female	Reference		Reference	
	Male	0.376 (0.242–0.582)	< 0.001	0.248 (0.132–0.465)	< 0.001

Age (years)	18–29	Reference		Reference	
	30–44	4.643 (2.796–7.709)	< 0.001	2.630 (1.244–5.558)	0.011
	45–59	9.364 (4.694–18.679)	< 0.001	4.793 (1.808–12.706)	0.002
	≥ 60	4.333 (1.977–9.497)	< 0.001	2.718 (0.898–8.233)	0.077

Education status	Uneducated	Reference		Reference	
	Educated	0.463 (0.278–0.771)	0.003	1.059 (0.547–2.048)	0.865

Marital status	Single	Reference		Reference	
	Married	5.194 (3.357–8.037)	< 0.001	3.313 (1.603–6.849)	0.001

Employment status	Unemployed	Reference		Reference	
	Employed	1.610 (1.079–2.403)	0.020	1.498 (0.843–2.662)	0.168

Smoking status	Nonsmokers	Reference		Reference	
	Smokers	0.435 (0.287–0.658)	< 0.001	0.657 (0.376–1.149)	0.141

Physical activity	No	Reference		Reference	
	Yes	0.699 (0.458–1.066)	0.096	0.762 (0.462–1.259)	0.289

Sleep disturbance	No	Reference		Reference	
	Yes	1.037 (0.689–1.562)	0.861	1.790 (1.076–2.977)	0.025

Diabetes	No	Reference		Reference	
	Yes	2.244 (1.071–4.702)	0.032	1.111 (0.456–2.711)	0.817

Hypertension	No	Reference		Reference	
	Yes	3.138 (1.389–7.090)	0.006	1.276 (0.477–3.414)	0.628

Hyperlipidemia	No	Reference		Reference	
	Yes	1.806 (0.910–3.586)	0.091	0.915 (0.393–2.130)	0.837

Ischemic heart disease	No	Reference		Reference	
	Yes	2.016 (0.679–5.985)	0.207	0.743 (0.201–2.749)	0.656

## 4. Discussion

Obesity is recognized as the most critical nutrition‐related condition and one of the most significant public health challenges because its prevalence continues to rise and its association with many negative health effects [4]. The national prevalence of obesity varies considerably across Middle Eastern countries, and regional comparisons are recommended to guide public health policy and epidemiological interpretation. In this study, the overall prevalence of overweight was 38.4%, which was higher than Iraq’s national prevalence (31.8%) [11] but lower than that found in Iran (40.97%) [13] and Turkey (61%) [14]. The overall prevalence of obesity was 24.3%, a rate that was lower than Iraq’s national estimates (33.9%) [11], Iran (30.13%) [13], and Turkey (32.1%) [14]. Furthermore, the overall prevalence of abdominal obesity was 72.9%, a rate that was higher than those reported in Iran (53.91%) [13] and Turkey (15.1%) [15].

The high prevalence of abdominal obesity in the current study may be partly explained by the high prevalence of overweight and obesity in our population (62.7%). Moreover, abdominal obesity can occur even among individuals with normal BMI, as BMI does not capture fat distribution or central adiposity. Additionally, the differences in prevalence observed between our study and those carried out in Iran and Turkey may be attributed to variations in population characteristics, including socioeconomic status and lifestyle patterns, as well as methodological differences such as sampling strategies and large sample size, which enhance the statistical power to detect significant associations. Additionally, obesity prevalence generally increases as countries become more developed [1].

Although no significant association was observed between gender and overweight and obesity, females had a higher prevalence of abdominal obesity (83.3% compared to 65.1% in males). The higher prevalence of abdominal obesity among females may be explained by social, structural, and gender‐related determinants rather than biological factors alone [16]. Age has consistently been found to be associated with higher body weight, and our study likewise found that increasing age was associated with higher BMI and waist circumference, likely reflecting metabolic changes, reduced physical activity, and lifestyle factors associated with aging [17]. The highest prevalence of overweight was observed among people aged 30–44 years, while those aged 45–59 had the highest prevalence of obesity and abdominal obesity. Similar findings have been reported in Iraq [11], Iran [13], Turkey [15], China [18], and the United States [19].

Participants with no formal education were significantly more likely to be obese (37.2% compared to 19.7%) and to have abdominal obesity (82.9% compared to 69.3%). However, this association was no longer statistically significant in the multivariate model. Other studies from Iraq [11] and Iran [13] have found an association between obesity and educational level, whereas no such association was observed in a study carried out in Turkey [15]. Individuals with higher educational levels may have greater awareness of the health consequences of overweight and obesity and are, therefore, more likely to adopt healthier lifestyles. Married individuals were significantly more likely to be overweight (44.4% compared to 30.3%), obese (33% compared to 12.8%), and to have abdominal obesity (86.4% compared to 55%) than single individuals. Our findings align with those previously reported in Iraq [11] and Turkey [15], but differ from those reported in Iran [13].

Employed participants in the univariate model had a significantly higher prevalence of overweight (42.3% compared to 33.6%), obesity (27.3% compared to 20.6%), and abdominal obesity (77.2% compared to 67.7%) than unemployed individuals. These findings are inconsistent with those reported in other studies from Iraq [11] and China [18]. However, these associations did not remain statistically significant after multivariable adjustment. Differences in occupational characteristics, physical activity levels, and population demographics may explain these inconsistencies in findings between studies. The higher prevalence of obesity among employed individuals may reflect sedentary occupational patterns, reduced opportunities for physical activity, and urban lifestyle characteristics that promote unhealthy dietary habits. In contrast, studies conducted in labor‐intensive or lower income settings have reported higher obesity among unemployed individuals, possibly due to socioeconomic stressors and unhealthy dietary patterns [20]. In the present study, smokers had lower odds of being overweight compared to nonsmokers. The relationship between smoking and obesity is complex. While smoking increases metabolic rate and reduces appetite, smoking cessation can lead to weight gain [21]. However, some studies have reported opposite results [17, 22].

Previous studies have found that physical inactivity and sleep disturbance are associated with overweight, obesity, and abdominal obesity. However, in the present study, only sleep disturbance exhibited a significant association with abdominal obesity [23, 24]. The absence of a significant association between overweight and obesity and physical inactivity may be attributed to the cross‐sectional design of the study, reliance on self‐reported lifestyle measures, and the use of simplified categorical variables, which could have limited the ability to detect true relationships. Obesity and abdominal obesity were significantly more prevalent among diabetic and hypertensive participants; however, these associations were no longer statistically significant in multivariable logistic regression analysis [24–26]. Although obesity is associated with an increased risk of cardiovascular diseases [4], the association with ischemic heart disease was not found to be significant in our study. This may be due to the relatively young study population, the cross‐sectional design, limited cases of clinically diagnosed CVD, or underreporting of events.

Although the 2015 Iraq STEPS survey provides valuable nationally representative estimates of overweight and obesity and explores several sociodemographic and behavioral correlates [11], our study differs in several methodological and contextual aspects that may explain variations in findings and support the relevance of our results. First, the national survey was based on a large multistage nationally representative sample of adults (*n* ˜ 3900), whereas our study focuses on an urban population in Duhok. This provides insights into regional patterns that may be overlooked in national‐level analyses. Second, the national survey primarily examined general obesity with normal/underweight as the reference category, while our study additionally provides a detailed analysis of abdominal obesity and uses normal weight as the reference category. Third, our study differed in the range of explanatory variables included in the analysis. Finally, the temporal difference between the datasets (2015 vs. 2025) may reflect changes in the epidemiology of obesity in Iraq. The findings of this study may help guide public health authorities in developing targeted screening and prevention strategies to address the growing burden of obesity in the region.

### 4.1. Strengths and Limitations

The current study is strengthened by its relatively large sample size, with the inclusion of both genders and adults from different age groups. Furthermore, the study provides province‐level estimates of overweight, obesity, and abdominal obesity in Duhok province, which could guide targeted public health initiatives tailored to the province’s needs. Moreover, we believe that this study will provide a landscape for future studies regarding the prevalence of overweight, obesity, and abdominal obesity in Iraq and their associated factors. Despite these strengths, the study has several limitations. First, the cross‐sectional design limits our ability to establish causal relationships. Moreover, some variables were self‐reported, introducing the potential for recall and misclassification bias. The study may also be subject to selection bias and residual confounding. Furthermore, several important related variables, including dietary habits, financial status, residential settings (urban vs. rural), family history, and medication use, were not included in the present study. Therefore, future studies should investigate these factors to provide a more comprehensive understanding of the factors associated with overweight, obesity, and abdominal obesity.

## 5. Conclusions

This population‐based study highlights a substantial burden of overweight, obesity, and abdominal obesity among adults in Duhok province, Kurdistan Region of Iraq. The identified associated factors provide valuable evidence for designing public health initiatives that prioritize more vulnerable groups. Public health programs should focus on educating people to adopt a healthier lifestyle through the promotion of physical activity, the avoidance of a sedentary lifestyle, and workplace health initiatives.

## Funding

No funding was received for this manuscript.

## Conflicts of Interest

The authors declare no conflicts of interest.

## Data Availability

The data that support the findings of this study are available from the corresponding author upon reasonable request.
